# NELFT Adult Autism Service Quality Improvement Project: Managing Demand, Capacity and Flow of Referrals for Adult Autism Assessments

**DOI:** 10.1192/bjo.2025.10385

**Published:** 2025-06-20

**Authors:** Fahima Khanom, Saras Saminathan

**Affiliations:** North East London NHS Foundation Trust, London, United Kingdom

## Abstract

**Aims:** The team joined the Royal College of Psychiatrists Quality Improvement (QI) Demand, Capacity and Flow (DCF) Collaborative. The aim was to increase the discharge rate to 19 per month following specialist assessment by June 2024.

**Methods:** Participants: NELFT Adult autism Service multi-disciplinary team (MDT), NELFT QI advisor, Directorate Business manager, referrers, autistic adult with living experience of the service and the provider improvement advisor.

Process: Using the NHS Quality Service Improvement and Redesign (QSIR) six-step approach (NHSE), the Learning Handbook (NHSE). A project driver diagram helped identify change ideas in the referral, screening, pre-assessment, assessment and post-diagnostic pathways.

Priorities: Change ideas in the screening, assessment and post-diagnostic stages were prioritised and three Plan, Do, Study, Act (PDSA) cycles. PDSA1, to increase the number of assessments conducted, PDSA2, to reduce screening time by removing first stage, PDSA3, to complete reports and discharge within 4 weeks of assessment.

**Results:** PDSA 1: Assessments

Data collected: assessment waiting time (years), appointments completed (Jan–Mar 2023).

Assessment waiting time from 3+ years to 2 years.

Assessments completed from 6 (Jan–Mar 2023) to 20 (Apr–Jun 2024).

PDSA 2: Screening

Data collected: time referral screening in meetings (minutes), adding to waiting list from meeting (days), adding to waiting list from referral (days), Qpack postage (days).

Referral received to client being added to waiting list in days: 42.4 to 37.5.

Average days between referral meeting and being added to waiting list: 51.5 to 1.7.

Time to screen referrals in meetings (per referral, sample of 20): 16 minutes to 10 minutes.

Referral to Qpack posted: 26 to 3 days (sample of 20).

PDSA 3: Post-Assessment

Data collected: additional appointments needed (number), time to write report (hours).

Number of additional appointments needed following assessments: 1.8 to 1.6.

Time to write reports from 5.5 hours to 4.5 hours.

**Conclusion:** These results show that DCF has increased across the pathways, but further PDSAs i.e. digitalising reporting need to be implemented to achieve the overall aim. The processes highlighted some of the challenges such as client complexities, maintaining staff morale and adjustment to change. There were also some unintended consequences such as the impact of improving one part of the pathway creating blockages in another.

Opportunities for learning from collaboration with key partners such as clients and referrers has been positive and inspired a more co-produced and creative approach to the methodology. The service will continue to utilise the PDSA cycles to test change new ideas and the QSIR framework to continually improve DCF.

